# Soil moisture remote sensing using SIW cavity based metamaterial perfect absorber

**DOI:** 10.1038/s41598-021-86194-2

**Published:** 2021-03-30

**Authors:** Majid Amiri, Mehran Abolhasan, Negin Shariati, Justin Lipman

**Affiliations:** 1 School of Electrical and Data Engineering, University of Technology Sydney, Sydney, NSW 2007 Australia; 2 Food Agility CRC Ltd, 81 Broadway, Ultimo, NSW 2007 Australia

**Keywords:** Environmental sciences, Engineering, Physics

## Abstract

Continuous and accurate sensing of water content in soil is an essential and useful measure in the agriculture industry. Traditional sensors developed to perform this task suffer from limited lifetime and also need to be calibrated regularly. Further, maintenance, support, and deployment of these sensors in remote environments provide additional challenges to the use of conventional soil moisture sensors. In this paper, a metamaterial perfect absorber (MPA) based soil moisture sensor is introduced. The ability of MPAs to absorb electromagnetic signals with near 100% efficiency facilitates the design of highly accurate and low-profile radio frequency passive sensors. MPA based sensor can be fabricated from highly durable materials and can therefore be made more resilient than traditional sensors. High resolution sensing is achieved through the creation of physical channels in the substrate integrated waveguide (SIW) cavity. The proposed sensor does not require connection for both electromagnetic signals or for adding a testing sample. Importantly, an external power supply is not needed, making the MPA based sensor the perfect solution for remote and passive sensing in modern agriculture. The proposed MPA based sensor has three absorption bands due to the various resonance modes of the SIW cavity. By changing the soil moisture level, the absorption peak shifts by 10 MHz, 23.3 MHz, and 60 MHz, which is correlated with the water content percentage at the first, second and third absorption bands, respectively. Finally, a $6 \times 6$ cell array with a total size of $312 \,\hbox {mm} \times 312 \,\hbox {mm}$ has been fabricated and tested. A strong correlation between measurement and simulation results validates the design procedure.

## Introduction

Landy introduced metamaterial perfect absorbers (MPA) in 2008^1^. This specific classification of FSS metamaterial absorbs the incident waves with near-unity efficiency. The first reported MPA structure consisted of a dielectric substrate, resonator on top, and another metal layer at the substrate’s backside. The resonator provides the electric response of MPA facing incident electromagnetic waves. Hence, the resonator’s shape and structure specify the different electric behavior targeted in an application. The interaction of the resonator and metallic layer on two substrate sides provides the structure’s magnetic response. Having described the MPA responses, different modifications could be applied to conventional MPA. For instance, a multi wideband absorption using multi resonators^2,3^, and multi-layer structure^4–6^ have been reported in several articles. The initial purpose of conventional MPA was replacing heavy, expensive, and low efficiency traditional absorbers such as Ferrit and high loss sheets. Beyond the early implementations, MPA’s capabilities with different absorption characteristics combined with a low profile and lightweight enable many new applications. Undesired signals absorbers^7–10^, thermal emitters^11,12^, terahertz applications^13–15^, optical switches^16,17^, energy harvesters^18,19^ and sensors^4,20^ are some of the popular applications of MPAs. It is worth noting that high absorption efficiency is a common requirement in all mentioned applications.

Sensors are a crucial part of modern technology in a wide variety of applications such as medicine, imaging, and agriculture. MPAs are a promising candidate to implement highly sensitive sensors. This is due to their flexibility to work at a wide range of frequency spectrum. A large number of MPAs have been introduced as a sensor in terahertz frequencies^21–24^. However, several restrictions, such as the complicated fabrication process, and the high cost of stable material in the terahertz spectrum, make implementing these sensors challenging. Moreover, the integration of terahertz sensors with other electrical devices is a further challenge due to the differences in size and operating frequency.

Microwave MPA sensors can overcome the above challenges. Several methods have been investigated to design MPA sensors at the microwave frequency spectrum. Implementing microfluidics channels in the structure has been reported in several articles. The channels are filled by injecting liquid or gas. It changes the substrate properties based on effective medium theory^25^. Consequently, the changes in resonance frequency are taken as a sensing parameter. It should be noted that the MPA needs to be calibrated prior to the sensing process. The channels are created in the additional layer attached to the MPA structure. Polydimethylsiloxane (PDMS) is a common material to make an extra layer that includes the channels^26,27^. The structures, which take advantage of this method are mostly complicated and need one or two connections for sensing. These characteristics could limit reported MPA sensors in large scale applications.

Another reported technique to use MPA as a microwave sensor is adding an analyte layer to the MPA structure^28–30^. In this method, an air gap can be added between the substrate and metal layer^31^ or between two substrates^32,33^. Like the previous method, the combination of the dielectric substrate and the sample provides an effective medium with a different permittivity ($\varepsilon _{eff}$). However, fabricating a multi-layer structure, including an air layer to place the sample in the metamaterial structure, is challenging.

In this paper, a cavity structure has been implemented to design a unit cell of MPA. The cavity has been widely used in various applications, such as antennas and filters, to improve their characteristics^34–38^. The capability of trapping electromagnetic signal and high Q-factor makes the cavity resonator a promising candidate for MPA structure in sensing applications. However, the fabrication complexity and 3D structure of cavity structure are challenges to implementing cavity MPA. Substrate integrated waveguide (SIW) technology can overcome the mentioned challenges of traditional cavities. Additional to the advantages of conventional cavities, the SIW structure offers a planar, compact, and low profile structure. Therefore, these properties lead to easing the integration of SIW cavity MPA with electrical circuits and passive components.

The high concentration of electric field energy in the SIW center cavity structure at its dominant resonance mode has been used in sensing applications. For instance, the proposed liquid material sensor in^39^ consists of two SIW cavities. The outer cavity reduces the size and increases the sensor’s Q-factor, while the middle SIW cavity, along with a microfluidic channel located on top of it, improves the sensing resolution. However, in the mentioned reports, the sensors need to be connected to the spectrum analyzer in transmission line mode to investigate the frequency shift and sense the targeted parameters. This requirement limits the usage of the sensor to invasive and laboratory applications. In another approach, the SIW cavity has been combined into the antenna to use as a sensor^40–42^. In this approach, the under-tested material is placed in a created slot in the middle of the cavity. Using the antenna structure eliminates having two connections for sensing procedure. Also, it facilitates remote sensing applications. However, similar to the filter sensor structure, antenna sensors need an electrical connection to them.

Precision agriculture requires monitoring and controlling environmental parameters such as temperature, weather conditions, and soil nutrition. One of the most impactful parameters in farming is remote monitoring the soil moisture. Besides the direct effect of soil moisture on growing the plants, it can stabilize the added fertilizer into the farm^43^. In this paper, a low-profile MPA with one layer structure has been investigated to perform in sensing application at microwave spectrum. Using MPA is a promising solution for remote sensing with no need for an electrical contact to design a non-invasive sensor. In this approach, the reflected waves from the MPA surface are used as sensing parameters that do not directly connect to the spectrum analyzer. Hence, it can facilitate wireless sensing by keeping the signal analyzer far away from the sensors. SIW cavity resonator has been used as a unit cell of MPA to design highly sensitive soil moisture sensors. Instead of using an extra PDMS layer, channels have been created in the SIW cavity. With the advent of various resonance modes in cavity structure, high-resolution sensing has been achieved by implementing the proposed MPA-based sensor. Three first mode resonance frequencies have been considered as sensing parameters. Despite dropping the absorption efficiency in higher modes, the resolution of the sensor increased accordingly. Furthermore, the proposed structure shows decent insensitivity facing electromagnetic waves with different incident angles and is polarization angle insensitive.

## MPA principles and unit cell design

The main principle to design an MPA is perfect impedance matching with air intrinsic impedance $Z_0=377$ $\Omega$. The perfect impedance matching guarantees the minimum wave reflection from structure surface ($\Gamma (\omega )=|S_{11} |^2 \approx 0$). However, minimum wave transmission is also required to have perfect absorption characteristics. Due to use of a metal film on the back side of the substrate the transmission coefficient of MPA is zero ($T(\omega )= |S_{21} |^2=0$). Considering these two parameters, absorption efficiency is obtained as Eq. (1):

1 $$\begin{aligned} A(\omega )=1-\Gamma (\omega )-T(\omega )=1- |S_{11} |^2 - |S_{21} |^2 = 1- |S_{11} |^2 \end{aligned}$$

In perfect impedance matching, the real and imaginary parts of $Z(\omega )$ have to be equal to 1 and 0, respectively. In another approach, the absorption ratio can be calculated based on the difference between $Z_{in}$ of MPA and intrinsic air impedance, according to the Eq. (2).

2 $$\begin{aligned} A(\omega )=1-\Gamma (\omega )=1-\bigg \vert {{\frac{Z_{in}(\omega )-Z_0}{Z_{in}(\omega )+Z_0}}}\bigg \vert ^2 \end{aligned}$$

where the input impedance of MPA ($Z_{in} (\omega )$) is calculated based on effective permittivity and permeability of MPA. These two parameters are a function of electric and magnetic responses at MPA resonance frequency. Input impedance of MPA can be calculated based on S-parameters of structure^44^ utilizing Eq. (3):

3 $$\begin{aligned} Z_{in}(\omega )=\sqrt{\frac{\mu _{eff}(\omega )}{\varepsilon _{eff}(\omega )}}=\sqrt{\frac{(1+S_{11}(\omega ))^2-S_{21}^2(\omega )}{(1-S_{11}(\omega ))^2-S_{21}^2(\omega )}} \end{aligned}$$

### Cavity resonator

A rectangular cavity is a short circuit ended rectangular waveguide. The reflection of the electromagnetic waves from conductive walls leads to creating a standing wave inside the cavity. The dimensions of the cavity resonator specify its characteristics. Different modes and mode numbers of electromagnetic waves ($TE_{mnp}$ and $TM_{mnp}$ ) can be resonated in the rectangular cavity. The resonance frequency of the cavity is calculated according to the Eq. (4)^45^:

4 $$\begin{aligned} f_{mnp}=\frac{C}{2\sqrt{\varepsilon _{r} \mu _{r}}} \sqrt{\left( \frac{m}{L}\right) ^2+\left( \frac{n}{H}\right) ^2+\left( \frac{p}{W}\right) ^2} \end{aligned}$$

where $C=3\times 10^{11}$ mm/s and $\varepsilon _r$ and $\mu _r$ are permittivity and permeability of dielectric inside the cavity. Moreover, W, L, and H are the width, length, and height of the cavity. It should be noted that the obtained resonance frequencies are for complete rectangular structure and adding slots on the walls lead to shifting the resonances of different mode numbers. In this paper, the final structure is to be used as a sensor. Hence, etching slots on the cavity walls and substrate facilitate merging the sample into the structure and creating a new effective area with different permittivity and permeability based on effective medium theory. Various properties of the located sample in the slots leads to changing the MPA absorption characteristics.

**Figure 1:**
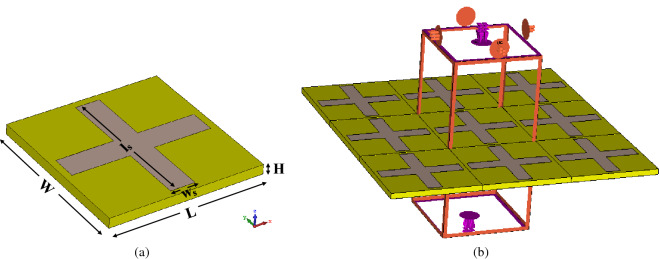
(**a**) Cavity resonator MPA unit cell , (**b**) Numerical setup to analyse MPA structure.

Figure 1a shows the layout of the cavity unit cell. Fr-4 with thickness (H) of 3.2 mm has been implemented as a substrate with dielectric constant $\varepsilon _r=4.3$ and loss tangent $tan \delta =0.014$. It has been used with two aspects: first, making the structure low-profile; and second, creating a firm structure that can last for a long time. Two perpendicular slots have been etched on the top wall of the cavity resonator. This layout of slots guarantees the resonating of the cavity facing both TE and TM polarized waves. Moreover, the symmetric structure is a crucial requirement for MPA to be polarization angle insensitive. The design process has been performed using CST Studio 2019 (Version 2019.07). As illustrated in Fig. 1b, a unit cell boundary condition is applied to a unit cell to model the metamaterial structure. Moreover, the Flouque port has been applied to analyse the proposed design under different polarization and incident angles of electromagnetic waves in TE and TM modes.

**Figure 2:**
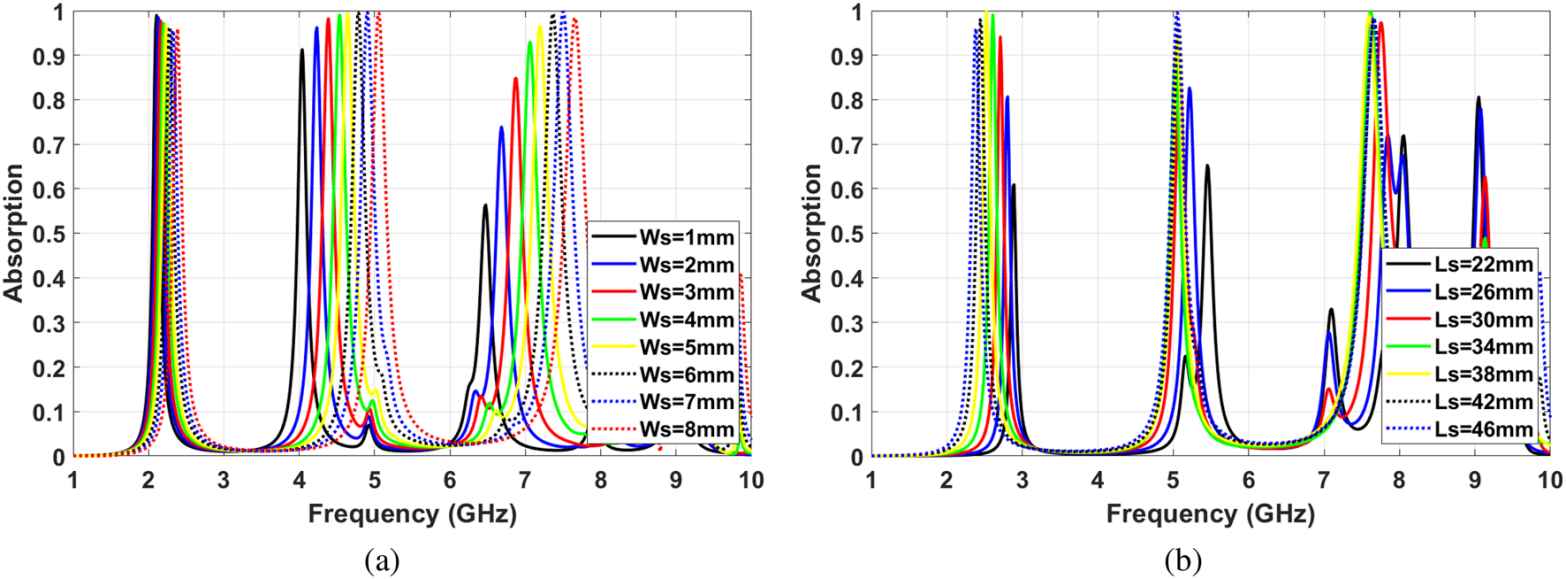
(**a**) Effect of changing slots width , (**b**) Effect of changing slots length.

Based on Eq. (4), in case of a much smaller height (H) of the cavity compared to width (W) and length (L), $TE_{m0n}$ modes are resonated in the cavity. Exciting the cavity using slots in the middle of the wall leads to create two electromagnetic zones on two sides of the slot. Consequently, the even modes resonate in the cavity. Moreover, some hybrid modes that are a combination of modes can resonate in the cavity. The dimension of the slot has a significant effect on the resolution of the proposed sensor. Figure 2 shows the absorbance of cavity MPA by varying the slot width (W) and Length (L), respectively. The slot size has a direct effect on matching the input impedance of MPA with intrinsic air impedance. As can be seen in Fig. 2a, widening the slot leads to shifting resonance frequency forward. This is due to decreasing equivalent capacitance of the structure followed by reducing the conductive area. Furthermore, the intensity of electromagnetic waves on two edges of slots is weakened by increasing the distance between them. Therefore, the equivalent capacitance decreases for wider slots due to the lower electrical charge on edges.

Changing the slot length leads to absorption frequency shift, as shown in Fig. 2b. Increasing the slots’ length leads to a smaller metallic surface that is one of the effective parameters to increase the equivalent capacitance. However, degrading the edges’ length causes a significant drop in the amount of electrical charge on the metallic layer. This phenomenon decreases the unit cell’s equivalent capacitance and shifts the resonance frequency upward as a consequence. It should be mentioned that slots’ lengths have been kept constant and equal to $l_s=46$ to investigate the effect of their width. Similarly, to illustrate the impact of slots’ width, their lengths have been kept equal to $w_s=8$.

All resonance modes can be used as a sensing parameter. Nevertheless, the higher mode resonances show more sensitivity due to the more peak points in e-field distribution. The cavity dimensions have been chosen to keep the targeted resonance modes at the commercial frequency spectrum. Both the width (W) and length (L) have been chosen equal to 50 mm as the cavity’s initial dimension. Based on the Eq. (4), the resonance frequencies of first, second, and third modes are at 2.2 GHz and 4.9 GHz 7.5 GHz for a cavity without slots.

### Substrate integrated waveguide structure

**Figure 3:**
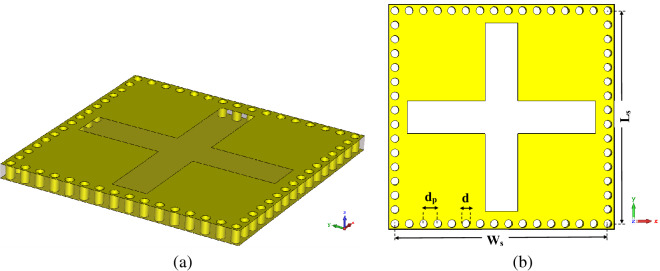
(**a**) SIW cavity resonator structure , (**b**) SIW cavity resonator dimensions.

**Figure 4:**
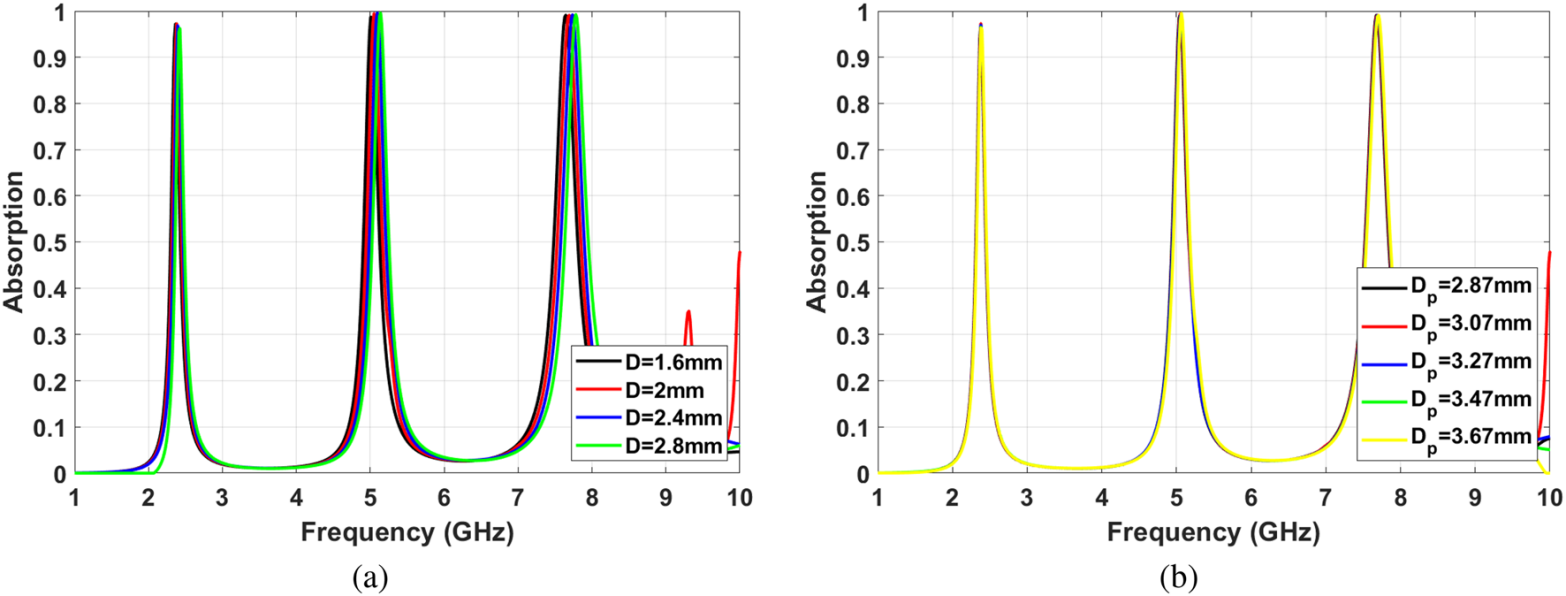
The effects of via hole parameters on absorption characteristics (**a**) Hole’s diameter, (**b**) Hole’s distance.

The conventional cavity can be modified to a planer and low-profile structure using the SIW technology, as shown in Fig. 3. Placing the metallic via instead of sidewalls of the cavity leads to dropping the propagation of surface waves. Hence, power leakage is reduced, and edge diffraction effects are suppressed. Moreover, using SIW technology improves the concentration of the electromagnetic wave in the cavity. This phenomenon enhances the practicality of proposed MPA in sensing applications. The following relations have to be considered to satisfy the least leakage of electromagnetic waves from SIW cavity walls as well as optimizing the number of hole vias^46^:

5 $$\begin{aligned} d< & {} \frac{\lambda }{5} \end{aligned}$$

6 $$\begin{aligned} d_p< & {} 2d \end{aligned}$$

where d is the hole via diameter, $d_p$ is center to center distance of to neighbor hole via, see Fig. 3b. The effects of changing hole’s diameter (*d*) and distance ($d_p$) considering the Eqs. (5) and (6) have been investigated. The results are shown in Fig. 4a,b. It should be noted that the hole’s distance must be chosen by considering the available space to have complete via hole. As can be seen, these two parameters’ effects are not significant, if they satisfy Eqs. (5) and (6).

Moreover, Eq. (4) needs to be tailored to obtain SIW cavity resonance. Due to circular shape of hole vias the width (W) and length (L) in Eq. (4) have to be replaced by effective width ($W_{eff}$) and effective length ($L_{eff}$), as calculated below^47^:

7 $$\begin{aligned} L_{eff}= & {} L_c-1.08\frac{d^2}{d_p}+0.1\frac{d^2}{L_c} \end{aligned}$$

8 $$\begin{aligned} W_{eff}= & {} W_c-1.08\frac{d^2}{d_p}+0.1\frac{d^2}{W_c} \end{aligned}$$

where $L_c$ and $W_c$ are the length and width of the SIW cavity representing center to center spacing of corner vias. These parameters are calculated using the Eqs. (7) and (8) by considering effective length ($L_{eff}$) and width ($W_{eff}$). To avoid changing cavity resonances after adding the via holes ($L_{eff}=L$) and ($W_{eff}=W$).

## MPA simulation results

**Figure 5:**
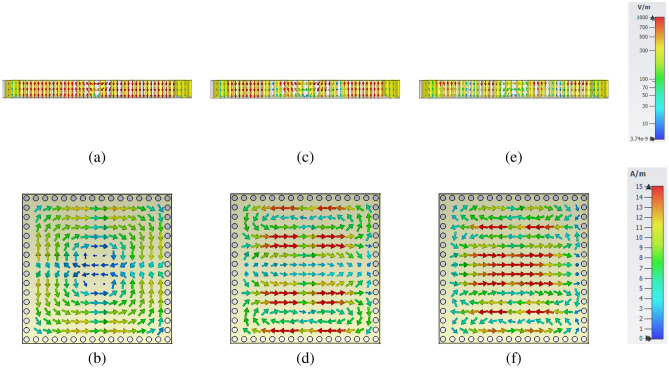
Electromagnetic fields facing TE polarized incident waves (**a**) E-field distribution at $f=2.4$ GHz ($TE_{102}$) , (**b**) H-field distribution at $f=2.4$ GHz ($TE_{102}$), (**c**) E-field distribution at $f=5$ GHz (Hybrid mode of $TE_{102}$ and $TE_{104}$) , **d** H-field distribution at $f=5$ GHz (Hybrid mode of $TE_{102}$ and $TE_{104}$) (**e**) E-field distribution at $f=7.7$ GHz ($TE_{104}$) , (**f**) H-field distribution at $f=7.7$ GHz ($TE_{104}$).This figures have been created by CST Studio 2019 Version 2019.07.

Table 1 represents the dimensions of the proposed unit cell to excite the first three modes of SIW cavity at 2.4 GHz, 5 GHz, and 7.7 GHz, based on previously described equations. Before implementing the SIW cavity MPA as a sensor, the proposed MPA’s characteristics need to be investigated facing different incident waves, including TE and TM polarized waves. The E-field and H-field distributions of the structure at resonance frequencies facing TE polarized incident waves are showing in Fig. 5, so as to provide better insight into the mechanism of the final structure and explain the resonant mode numbers. The structure’s dimension leads to the exciting of $TE_{mnp}$ facing both TE and TM polarized incident waves. Figure 5a,b show the E-field and H-field distributions of $TE_{102}$ mode at $f=2.4$ GHz. These figures illustrate two electromagnetic zones at the different centers by slot, are out of phase, and have equal amplitude for electric and magnetic fields.

Additional to common modes, one hybrid mode is excited in the proposed SIW cavity at f $=$ 5 GHz. Figure 5c,d show the electric and magnetic field distributions in the unit cell of MPA, respectively. Two completely separated out of phase electromagnetic zones is represented by $TE_{102}$ modes. Moreover, two smaller out of phase electromagnetic zones are created. This mode is a hybrid mode created by combining $TE_{102}$ and $TE_{104}$ modes. Furthermore, $TE_{104}$ mode resonates at f $=$ 7.7 GHz in the proposed structure. Figure 5e shows the E-field distribution of structure in this mode. There are four different electric zones with an alternative change in their phases. Also, these four individual zones are seen in H-field distribution; see Fig. 5f.

**Table 1:** The optimum values of unit cell dimension.

Parameter	L	W	$l_s$	$w_s$	$L_s$	$W_s$	$d_p$	d
Value	50 mm	50 mm	46 mm	8 mm	51.263 mm	51.263 mm	3.47 mm	2 mm

**Figure 6:**
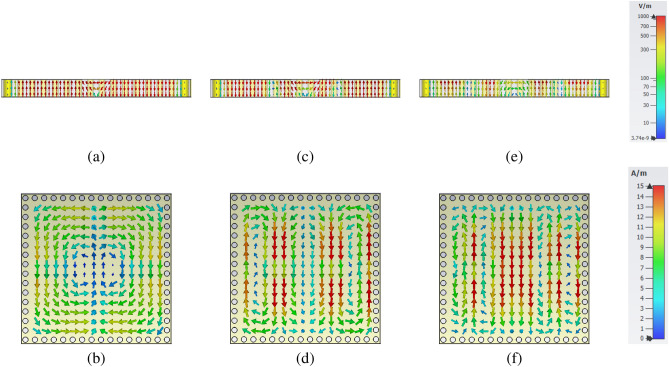
Electromagnetic fields facing TM polarized incident waves (**a**) E-field distribution at $f=2.4$ GHz ($TE_{201}$) , **b** H-field distribution at $f=2.4$ GHz ($TE_{201}$), (**c**) E-field distribution at $f=5$ GHz (Hybrid mode of $TE_{201}$ and $TE_{401}$) , (**d**) H-field distribution at $f=5$ GHz (Hybrid mode of $TE_{201}$ and $TE_{401}$) (**e**) E-field distribution at $f=7.7$ GHz ($TE_{401}$) , (**f**) H-field distribution at $f=7.7$ GHz ($TE_{104}$). This figures have been created by CST Studio 2019 Version 2019.07.

The SIW cavity unit cell and perpendicular slot’s dimension leads to similar E-filed and H-field concentration patterns and the other slot (Y-axis) for TM polarized incident waves. It means that $TE_{201}$, hybrid mode of $TE_{201}$ and $TE_{401}$, and $TE_{401}$ modes are excited in the SIW cavity facing TM polarized incident waves. Figure 6 shows the E-field and H-field distribution facing TM polarized incident waves at resonance frequencies.

As shown in Figs. 5 and 6, the waves enter via slots and distribute along the cavity symmetrically. The number of peak points along each x and y-axis specifies the mode’s order for m and n. However, the E-field’s centering around the slots facing different polarized incident waves is common for all mode numbers. This field concentration leads to creating a dipole structure on the vertical or horizontal slot depending on the incident wave polarization. Based on the Maxwell equation, the electric field creates an electrical charge on the metallic surface. The electrical charge interaction on the surface and bottom layer create a magnetic dipole inside the SIW cavity resonator. Two edges of slot and metallic surfaces on top and bottom of the cavity provide the structure’s capacitive behavior. On the other hand, rotating magnetic fields create surface currents that provide the structure’s inductive behavior. The coupling of these structures’ properties, which are due to the electric and magnetic responses, leads to a strong local resonance in each cavity resonator mode.

**Figure 7:**
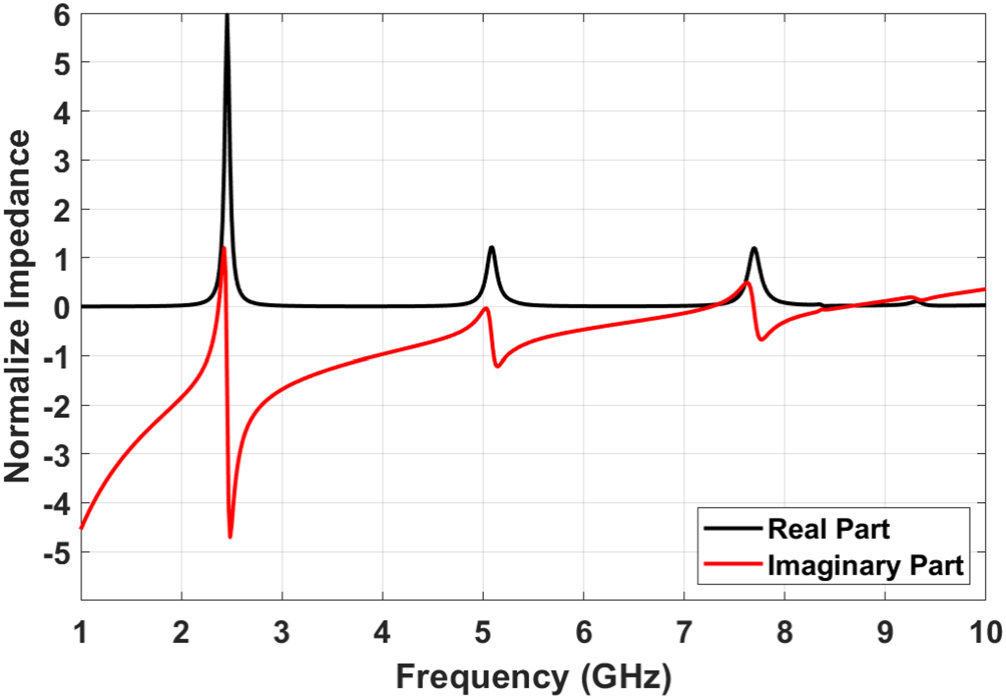
Input impedance of SIW cavity resonator.

The input impedance of final SIW cavity MPA has been obtained based on Eq. (3) and is shown in Fig. 7. It should be noted that the impedance graph has been normalize to air intrinsic impedance. As mentioned before, ideal absorptivity would be achieved if the real and imaginary parts of input impedance are 1 and 0, respectively. The input impedances of proposed MPA are $Z_{in1}=0.81+j0.19$, $Z_{in2}=0.91-j0.09$ and $Z_{in3}=1.1-j0.01$ at $f=2.4$ GHz, $f=5.05$ GHz and $f=7.65$ GHz, respectively. The small difference between $Z_{in}$ at resonance frequencies and $Z_0$ confirm the near unity absorption based on Eq. (1). The Q-factors are 26.4, 34.8 and 36.61 at resonance frequencies in order. Moreover, effective permeability and permittivity are two other important parameters to show the metamaterial behaviour. These parameters are calculated using the refractive index of structure^44,48^ based on Eq. (9):

9 $$\begin{aligned} n_{eff}(\omega )= \pm \left( \frac{1}{kL}\right) arccos\left( \frac{1-S_{11}^2+S_{21}^2}{2S_{21}}\right) \end{aligned}$$

**Figure 8:**
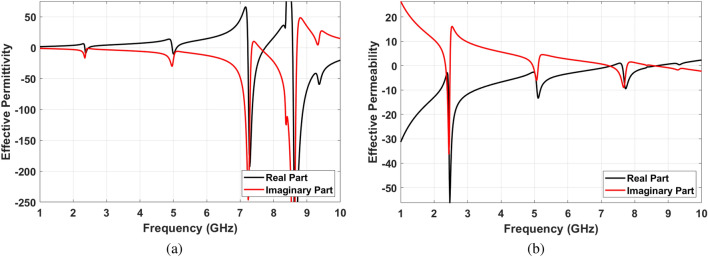
(**a**) Effective permittivity , (**b**) Effective permeability.

Where k and L are the wave wavelength and the unit cell size, respectively. By calculating the MPA impedance and effective refractive index, permeability and permittivity are defined in Eqs. (10) and (11), respectively. As can be seen in Fig. 8a,b, both effective permittivity and permeability are negative around resonance frequency ($\varepsilon _{eff}<0$ and $\mu _{eff}<0$).

10 $$\begin{aligned} \mu _{eff}(\omega )= & {} n_{eff}(\omega )Z_{eff}(\omega ) \end{aligned}$$

11 $$\begin{aligned} \varepsilon _{eff}(\omega )= & {} n_{eff}(\omega )/Z_{eff}(\omega ) \end{aligned}$$

**Figure 9:**
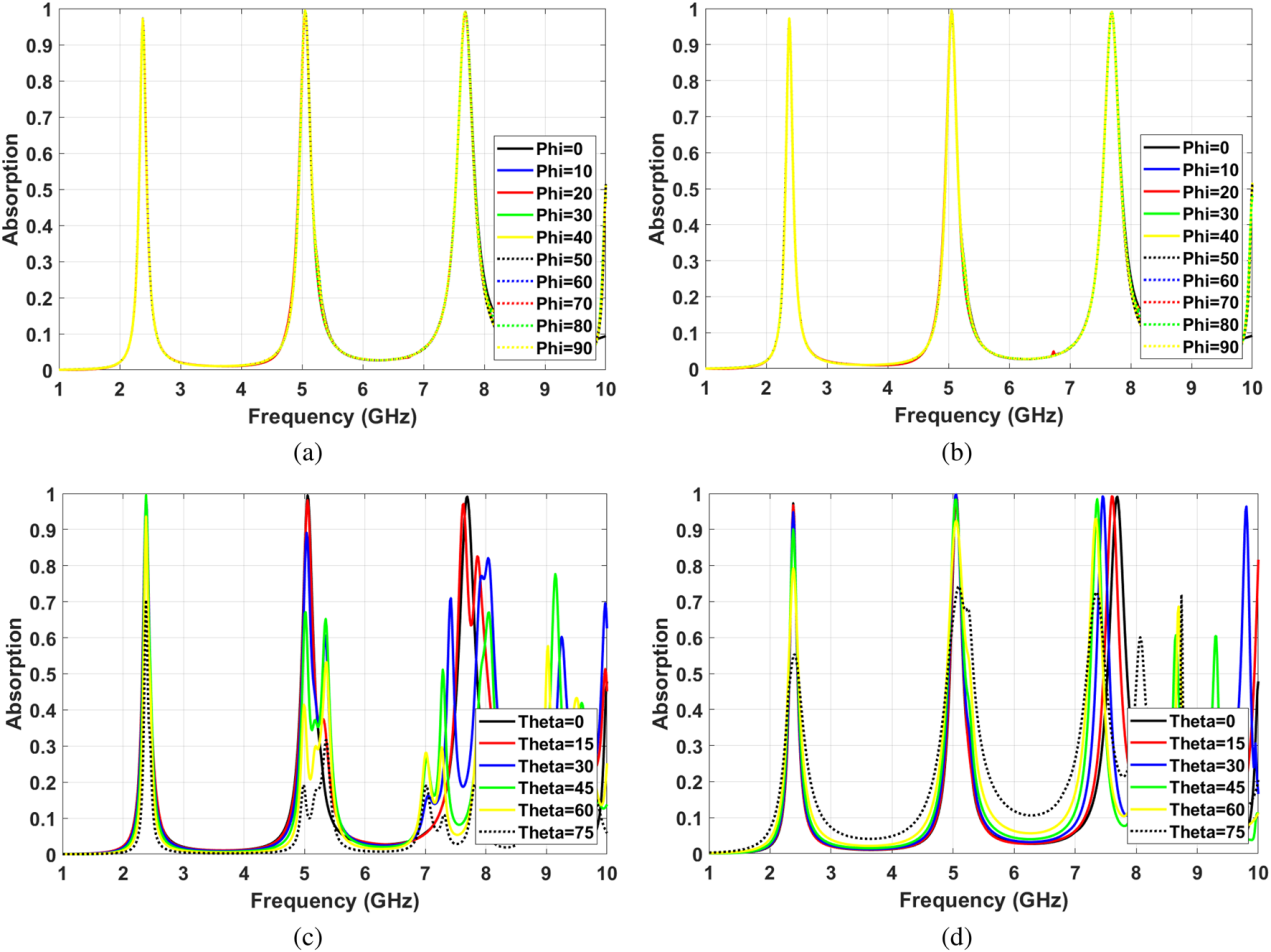
Angle insensitivity (**a**) Different polarization angles for TE waves, (**b**) Different polarization angles for TM waves, (**c**) Different incident angles for TE waves, (**d**) Different incident angles for TM waves.

The insensitivity of absorption characteristics facing various incident waves is an important parameter due to different polarization and incident angles. Some reported designs show a perfect insensitivity facing different polarization and incident angles for a broad spectrum^49,50^. However, having a high Q-factor followed by sharp resonance is crucial for sensing applications. The proposed SIW cavity MPA shows perfect insensitivity for changing polarization angles from $\phi =0$ to $\phi =90$ because of the symmetric shape along with high Q-factor at all resonance frequencies. Fig. 9a,b illustrate that the absorption characteristic remains steady for all polarization angles for TE and TM polarized modes. Also, It can be seen that the proposed structure is the polarization angle insensitive for all the mode numbers.

On the other hand, the insensitivity of absorption characteristics facing different incident angle shows various behavior for different model numbers. As Fig. 9c shows, for $TE_{102}$ mode (f $=$ 2.4 GHz), absorption ratio remains above 80% by increasing the incident angles up to $\theta =60 ^{\circ }$. Furthermore, the resonance frequency shows almost no shift by increasing the incident angles. Moreover, for hybrid mode (f $=$ 5 GHz), the absorption ratio is more than 60% for incident angles to $\theta =45^{\circ }$. Also, the absorption frequency shifts are negligible. Whereas, for $TE_{104}$ mode (f $=$ 7.7 GHz), the absorption ratio stays higher than 65% incident angles to $\theta =45^{\circ }$, while the absorption frequency shifts from 7.7 GHz to 8 GHz for this range of incident angle.

Facing TM polarized incident waves, the absorption stability for $TE_{102}$ (f $=$ 2.4 GHz) is slightly less than TE mode. However, the absorption ratio is higher than 80% at incident wave angles up to $\theta =60 ^{\circ }$ that is reasonable absorption insensitivity. Nevertheless, for hybrid mode (f $=$ 5 GHz), the absorption characteristics show better insensitivity facing TM waves compared to TE ones. The absorption ratios of hybrid mode at $\theta =60 ^{\circ }$ and $\theta =75 ^{\circ }$ are 92% and 74%, respectively. Meanwhile, the absorption frequency shows a negligible shift and is fairly stable at high incident angles up to $\theta =75 ^{\circ }$. As it can be seen in Fig. 9d the absorption ratios of $TE_{401}$ at $\theta =60 ^{\circ }$ and $\theta =75 ^{\circ }$ are 93% and 71%, respectively. However, the absorption frequency shifts by 400 MHz. Based on the represented results in Fig. 9c,d, the insensitivity for different incident angles is significant for both TE and TM modes at the first and second resonances. This is due to the strong magnetic response of the cavity resonator.

The soil moisture remote sensing setup can be implemented using controllable or random electromagnetic signals. In controlled scenarios, the position of the transmitter, receiver, and sensor are chosen. Hence, the incident angle can be easily set. However, the sensor should be insensitive enough for changing polarization and incident angles to handle implementation errors. On the other hand, random ambient electromagnetic signals are other candidates to be used in sensing applications. In this case, the sensor must be insensitive for changing incident and polarization angles. Therefore, the first two resonances are appropriate for both controlled sensing scenarios and uncontrolled sensing. On the other hand, the third resonance is not suitable for uncontrolled sensing scenarios due to the possibility of false sensor reading for the higher incident angle. However, the third mode’s high resolution can be implemented to sense multiple soil parameters such as salinity and nutrition simultaneously.

Converting the polarization instead of absorbing the incident signals can lead to sensor inaccuracy. To avoid this issue, polarization conversion ratio (PCR) has to be minimum. In more detail, the reflected waves from MPA structure consist co-polarized and cross-polarized components. Co-polarization reflection coefficients can be in x and y directions that are defined as $R_{xx}=|E_{rx}|/|E_{tx}|$ and $R_{yy}=|E_{ry}|/|E_{ty}|$. Furthermore, cross-polarization reflection coefficient are determined as $R_{xy}=|E_{rx}|/|E_{ty}|$ and $R_{yx}=|E_{ry}|/|E_{tx}|$. Where $E_{tx}$ and $E_{ty}$ are defined as the x-polarized and y-polarized components of transmitted waves. In addition, $E_{rx}$ and $E_{ry}$ are components of reflected waves. Considering described coefficients, PCR for TE and TM modes are calculated based on Eqs. (12) and (13).

12 $$\begin{aligned} PCR_{TE}= & {} \frac{R_{yx}^2}{R_{yy}^2+R_{yx}^2} \end{aligned}$$

13 $$\begin{aligned} PCR_{TM}= & {} \frac{R_{xy}^2}{R_{xx}^2+R_{xy}^2} \end{aligned}$$

**Figure 10:**
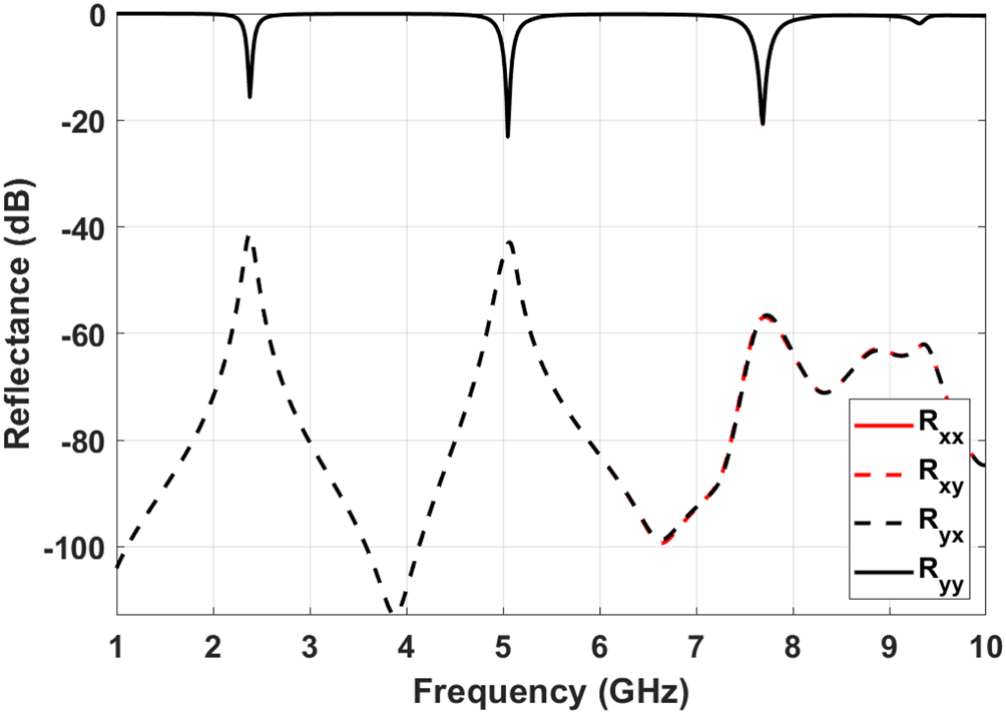
Co- and cross-polarization reflection.

Figure 10 shows the reluctance coefficients of proposed MPA, which are similar in x and y-direction due to the symmetrical structure. As can be seen, cross-polarization components are negligible even in resonance frequencies. The PCR of the structure facing TE and TM polarized incident waves are 0.0027, 0.0099, and 0.00024 at first, second, and third resonance frequencies. Near zero PCR illustrate the absorption capability of the proposed structure without polarization conversion.

### Channeling to create effective medium

**Figure 11:**
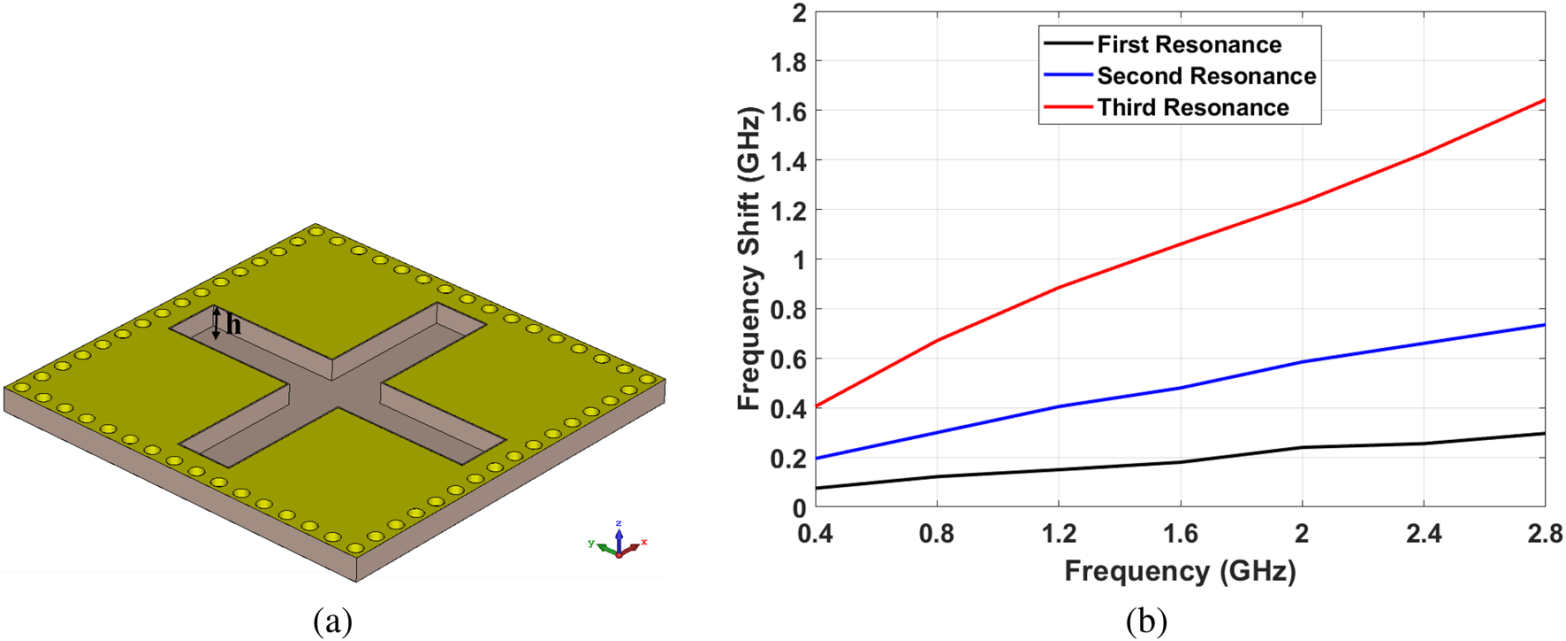
(**a**) Final unit cell after etching the channels , (**b**) The frequency shift by changing the soil moisture content from 0% to 30% for different depth of channels.

As mentioned before, adding an air gap as a sample holder impacts the fabrication complexity and practicality of structure in real-world scenarios. In this paper, perpendicular channels between the slots have been etched in the substrate. The sample can be easily placed in these channels, even for a large structure. Moreover, there is no need to add an extra layer to place the sample in this approach. Figure 11a shows the channeled unit cell of SIW cavity MPA. The combination of dielectric and soil in channels creates a new medium that is represented by effective permittivity and permeability ($\varepsilon _{eff}$ and $\mu _{eff}$). The slot’s rectangular layout leads to maximum soil sample interaction and electric field on slot’s edges. The symmetric pattern of the final structure leads to significant insensitivity facing different polarization and incident angles. Meanwhile, this shape of channels facilitates the maximum electromagnetic waves passing through the sample that leads to a higher resolution of the proposed sensor.

It is noteworthy that more volume of the sample leads to more shifts in absorption frequency. Hence, these channel’s depth is an important parameter to achieve the best performance that needs to be investigated with two aspects. One is the sensitivity of MPA that is increased by deepening the channels. The second consideration is the rigidity of structure in practical applications that decreases for deeper channels. Figure 11b shows the shift of absorption frequency by changing the soil’s water content from 0% to 30% for different channel depths. As can be seen, the sample’s effect on absorption characteristics increases for deeper channels. This is due to the bigger change in effective permittivity and permeability. It is also obvious from Fig. 9b that frequency shift increases at higher resonance modes. In the proposed MPA structure, the depth of the channels has been chosen as $d=2.8$ *mm*. The sensitivity is slightly compromised by not deepening the channel more to have a solid structure for real-world applications.

## Soil properties with different level of moisture

In this paper, sensing different levels of soil moisture has been targeted. Thus, soil properties with varying water content levels must be investigated prior to adding them to the structure. Permittivity ($\varepsilon _{soil}$) and loss tangent ($tan\delta$) are important characteristics of under test material in sensing process. The behaviour of material in electric field is described as Eq. (14)^45^:

14 $$\begin{aligned} \varepsilon _r=\varepsilon \;' _r + j \varepsilon \;''_r \end{aligned}$$

where $\varepsilon \;' _r$ is the dielectric constant that represent the capability of the storing electric field , while $\varepsilon \;''_r$ is the loss factor that shows the ratio of electric field loss in the material. Using these parameters, the loss tangent is defined according to the Eq. (15):

15 $$\begin{aligned} tan \delta = \frac{\varepsilon \;'' _r}{\varepsilon \;'_r} \end{aligned}$$

In^51^, the properties of different soil types with various moisture levels have been investigated at different spectrums. Considering Eqs. (14) and (15), sand, loam, and clay soil characteristics with various moisture levels at 4 GHz spectrum have been represented in Table 2. It should be noted that the soil properties at 2.4 GHz and 5 GHz can be estimated by their values at 4 GHz due to minor changes in properties in this range of frequency^52^.

**Table 2:** The characteristics of various types of soil with different levels of moisture.

Soil type	Moisture level	Dielectric constant	$tan \delta$
Sand	0%	3.0	0.033
	5%	3.9	0.153
	10%	5.3	0.2.7
	15%	7.2	0.236
	20%	9.9	0.232
	25%	13.2	0.227
	30%	16.9	0.224
Loamy	0%	3.0	0.033
	5%	3.8	0.131
	10%	4.8	0.187
	15%	6.3	0.222
	20%	8.5	0.235
	25%	11.6	0.215
	30%	15.4	0.207
	35%	19.2	0.198
	40%	23	0.191

## Sensing capability of proposed sensor

**Figure 12:**
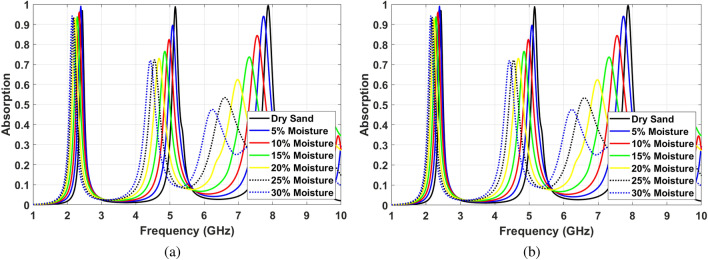
Sensing efficiency of the proposed MPA for different moisture levels in sand soil (**a**) Facing TE polarized incident waves, (**b**) Facing TM polarized incident waves.

The absorption ratio and frequency of SIW cavity MPA show different changes for various depths of channels at f $=$ 2.4 GHz, f $=$ 5 GHz, and f $=$ 7.7 GHz. Figure 12a shows these changes facing TE polarized incident waves. In this regard, the proposed structure’s sensing capability needs to be investigated individually for each frequency. The first resonance frequency ($TE_{101}$) shifts from 2.4 to 2.1 GHz when soil moisture content increases from 0 to 30%. This shift in absorption frequency can be considered as 10 MHz per one percentage of moisture content. The absorption ratio varies as the soil moisture changes. This can then be considered as an additional parameter when sensing soil moisture. However, for this mode, the absorption change is negligible within the 300 MHz shift of frequency.

At f $=$ 5 GHz ($TE_{103}$ mode), the absorption frequency shifts 700 MHz downward for 30% change in soil moisture, which translates to 23.3 MHz per one percentage of water content. Furthermore, the absorption ratio of the proposed structure at the second absorption band decreases almost linearly from 99.8 to 82% when moisture changes 30%. At f $=$ 7.8 GHz ($TE_{105}$ mode), the frequency shifts 1.8 GHz and absorption ratio drops from 99.9 to 48%. These parameters can be translated to a 60 MHz frequency shift and 1.8% percentage of absorption ratio per one percent of soil moisture level. Therefore, the fifth mode shows better sensitivity to the level of water content in the soil. Due to the dimension and the unity ratio of width and length of the proposed cavity-based MPA, similar behavior can be seen facing TM polarized incident waves, see Fig. 12b. In this case, $TE_{101}$, $TE_{101}$ and $TE_{101}$ modes are excited at f $=$ 2.4 GHz, f $=$ 5 GHz and f $=$ 7.8 GHz, respectively.

**Figure 13:**
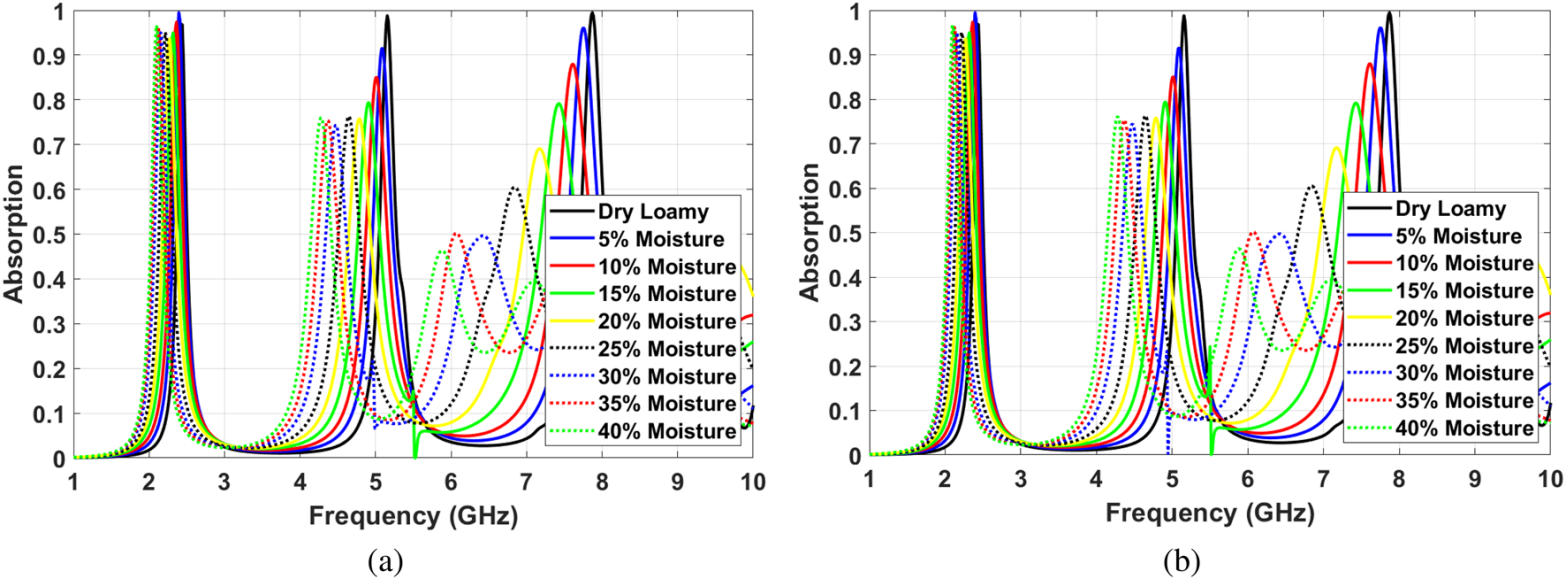
Sensing efficiency of the proposed MPA for different moisture levels in Loamy soil (**a**) Facing TE polarized incident waves, (**b**) Facing TM polarized incident waves.

The proposed structure can be implemented to sense moisture content in different types of soil. Figure 13a,b shows the absorption characteristics of SIW cavity MPA by adding Loamy soil with different levels of moisture in channels facing TE and TM polarized incident waves, respectively. As can be seen in these figures, the absorption characteristics show similar behavior for different soil types. It means the absorption ratio drops by increasing the soil moisture level, and the absorption frequency shifts downward. Moreover, the sensitivity of MPA increases for higher resonance order.

Facing TE polarized incident waves, at the first resonance frequency, the absorption ratio is almost unchanged and higher than 95% for various levels of moisture, as shown in Fig. 13a. However, the absorption frequency shift from 2.4 to 2.1 GHz when the soil moisture changes from 0 to 40%. It means almost 7.5 MHz per percentage of soil moisture. At the second resonance frequency, the absorption frequency shifts from 5.13 to 4.23 GHz by adding Loamy soil moisture levels up to 40%. This shift means 22.5 MHz change per percentage of soil moisture. Besides, the absorption ratio falls off from 99 to 76%. Similar to sensing the sand moisture level, the third absorption frequency shows the highest sensitivity. Dropping the absorption ration from 99.9 to 46% and shifting the absorption frequency from 7.9 to 5.8 GHz for changing soil moisture in the range of 0% and 40% provides a highly sensitive low-profile sensor. It should be noted that the sensing capability of the structure facing TM polarized incident waves is similar to TE mode, as can be seen in Fig. 13b.

Different methods have been implemented to design highly sensitive and robust soil moisture sensors. Table 3 has been provided to compare the capability of the proposed structure, commercial, and previously reported scientific sensors. This comparison covers various parameters, such as accuracy, resolution, longevity, cost, and maintenance requirement. As can be seen, the proposed sensor offers high resolution and the highest life expectancy. Also, unlike commercial sensors, the proposed structure can be fabricated and implemented at a much lower cost. In addition to the accuracy and resolution, deployment capability on a large scale is a crucial requirement in modern agriculture. Commercial sensors consist of a probe that interacts with the environment and an analyser that processes the probe’s output into presentable data. For large scale deployment, the sensor’s analyser has to be connected to a data transmission network. Implementing a compatible transmission network can be a challenging and expensive process requiring extra infrastructure. The proposed structure is able to perform wirelessly with no need for an attached analyser section. The analyser section includes a signal transmitter, a signal receiver, and a signal processor that can be placed at a distance from the sensors. The concept of wireless sensing with one data analyser unit for all sensing scenarios removes the need for a complicated data transmission network.

Radio frequency sensors found in scientific literature^27,28,39,40,42,53–57^ have been designed to sense solid or liquid analytes. The common consideration in design procedure among all reported structures is creating a specific place to add analyte into the sensor. Taking advantage of radio frequency behaviour facing different mediums, changing transmission, or reflection coefficients by introducing analyte is considered as a sensing parameter. The reported structures are low-profile and show reasonable sensitivity and longevity. However, these sensors are not suitable for remote sensing scenarios as they need a physical connection to add analyte or transmit RF signals through the structure. The proposed structure in this paper has been compared with reported sensors in the literature that used various methods in Table 4. The performance of sensors is defined by sensitivity. Various equations have been used to calculate this parameter. Herein, the average sensitivity of sensor ($S_{ave}=(\Delta f/f_0)/(\Delta \varepsilon /\varepsilon _{eff}$) has been considered to unify the sensing capability targeting different analytes^58^. Where $\varepsilon _{eff}$ is the maximum of $\varepsilon _r$ considering the variation in analyte’s concentration. As shown in Table 4, the sensitivity of the proposed sensor shows significant improvement compared to previously reported structures. It should be noted that the Table 4 comparisons are based upon simulation result to provide a fair comparison.

**Table 3:** Comparing different types of soil moisture sensor based on various methods.

Advantages	Category								
	Sensor type	Accuracy	Resolution	Longevity	Measurement zone	Large scale deployment	Cost	Maintenance	Connection
Commercial Sensor	TDR/TDT	Excellent	Excellent	20 years	< 1 cm from sensor	Hard and Expensive	High	Not Required	Required
	Capacitance/FDR	Satisfactory	Good	2 to 5 years	< 1 cm from sensor	Expensive	Low	Calibration	Required
	Standing Wave	Good	Excellent	20 years	2 cm from central needle	Expensive	Moderate	Calibration	Required
	Neutron Prob	Excellent	Excellent	20 years	30 cm diameter sphere	Expensive	High	Calibration	Required
Scientific Sensors	Metamaterial Filter^53–55^	Excellent	Satisfactory	Long(Depends on Material)	Small	Impossible	Low	Not Required	Required
	Multi-layer MPA with PDMS^26,27^	Excellent	Satisfactory	Long(Depends on Material)	Medium	Impossible	Moderate	Not Required	Required
	Multi-layer MPA Air-Gap^28–30^	Excellent	Satisfactory	Long(Depends on Material)	Medium	Impossible	Low	Not Required	Required
Proposed Sensor	Cavity-based MPA	Excellent	Excellent	Life-Time	Big	Easy and Inexpensive	Low	Not Required	Not Required

**Table 4:** Some reported MPAs for removing undesired signals applications.

Reference	Method	$f_0$ GHz	$\Delta f$	min $\varepsilon _r$	max $\varepsilon _r$	Sensitivity	Analyte	Concentration	Wireless sensing	Changing analyte
^42^	SIW Antenna	5.09	180MHz	5.22	75	3.8	Ethanol	5–100%	No	Required
^53^	SRR filter	1.895	110 MHz	8.4	79	6.5	Ethanol	0–100%	No	Required
^54^	CSRR filter	1.73	400 MHz	9.7	79	26.3	Ethanol	0–100%	No	Required
^55^	Resonator filter	10.94	1.04 GHz	4	44	10.45	Ethanol	0–100%	No	Required
^40^	SIW Antenna	4.4	400 MHz	5.4	75	9.8	Ethanol	0–100%	No	Required
^56^	MTM-based Filter	0.89	60 MHz	27.86	80	11.75	Ethanol	10–100%	No	Required
^39^	SIW Filter	1.59	40 MHz	21	79	3.42	Acetone	0–100%	No	Required
^27^	MPA with PDMS layer	9.49	1.09 GHz	4.5	68	12.3	Ethanol	0–100%	No	Required
^57^	MPA with PDMS layer	4.1	400 MHz	23	66	14.8	Ethanol	0–100%	No	Required
^28^	MPA with air gap	7.31	410 MHz	2.95	3.38	44.1	Mass Fraction	0–100%	Yes	Required
Proposed Structure	SIW Cavity MPA	2.4, 5.1, 7.7	300MHz, 700 MHz, 1.8 GHz	3	16.9	50.66, 55.62, 94.74	Soil Moisture	0–30%	Yes	Not Required

## Fabrication and measurement

**Figure 14:**
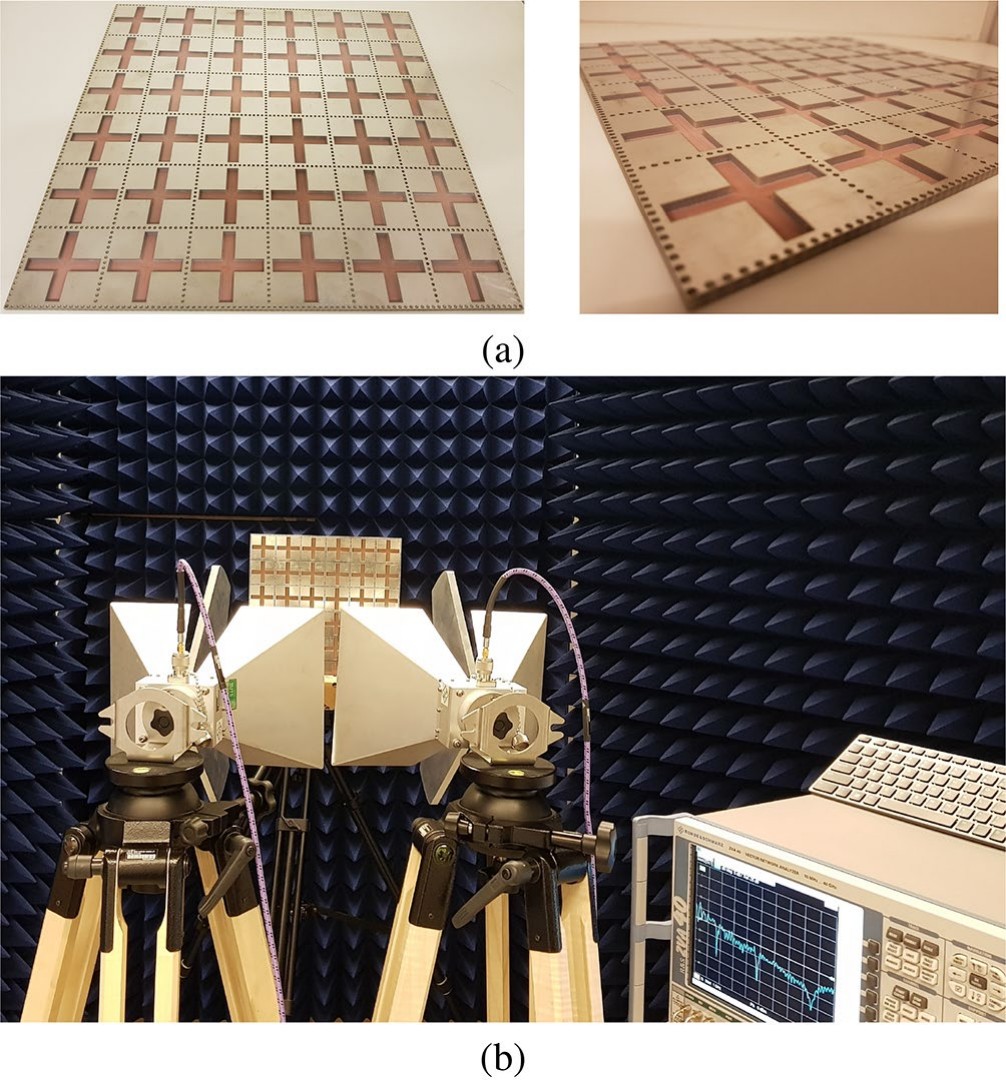
(**a**) Fabricated $6 \times 6$ MPA, (**b**) Implemented measurement setup.

To prove the design procedure and the simulation results, the final SIW cavity MPA has been fabricated in a $6 \times 6$ array structure that is shown in Fig. 14a. The plus shape slots have been etched on the copper layer on top of the FR-4 substrate. Then, the via holes and channels have been created in the structure. Two horn antennas are located one meter away from MPA to satisfy the far-field distance (more than10 $\lambda$). One of the antennas works as a transmitter, and the other one works as a receiver. Absorption characteristics for different polarization angles are measured by rotating reference antennas around the horizontal axis simultaneously. On the other hand, to change the incident angle, horn antennas are moved on a half-circle with a radius of one meter in opposite directions. Figure 14b shows the measurement setup. Prior to performing the measurements, the setup must be calibrated to specify the transmission coefficient’s base level between two antennas. In this regard, a metal plate with the same MPA size is placed instead of the final structure. These results are used to reduce the noise of measured characteristics.

**Figure 15:**
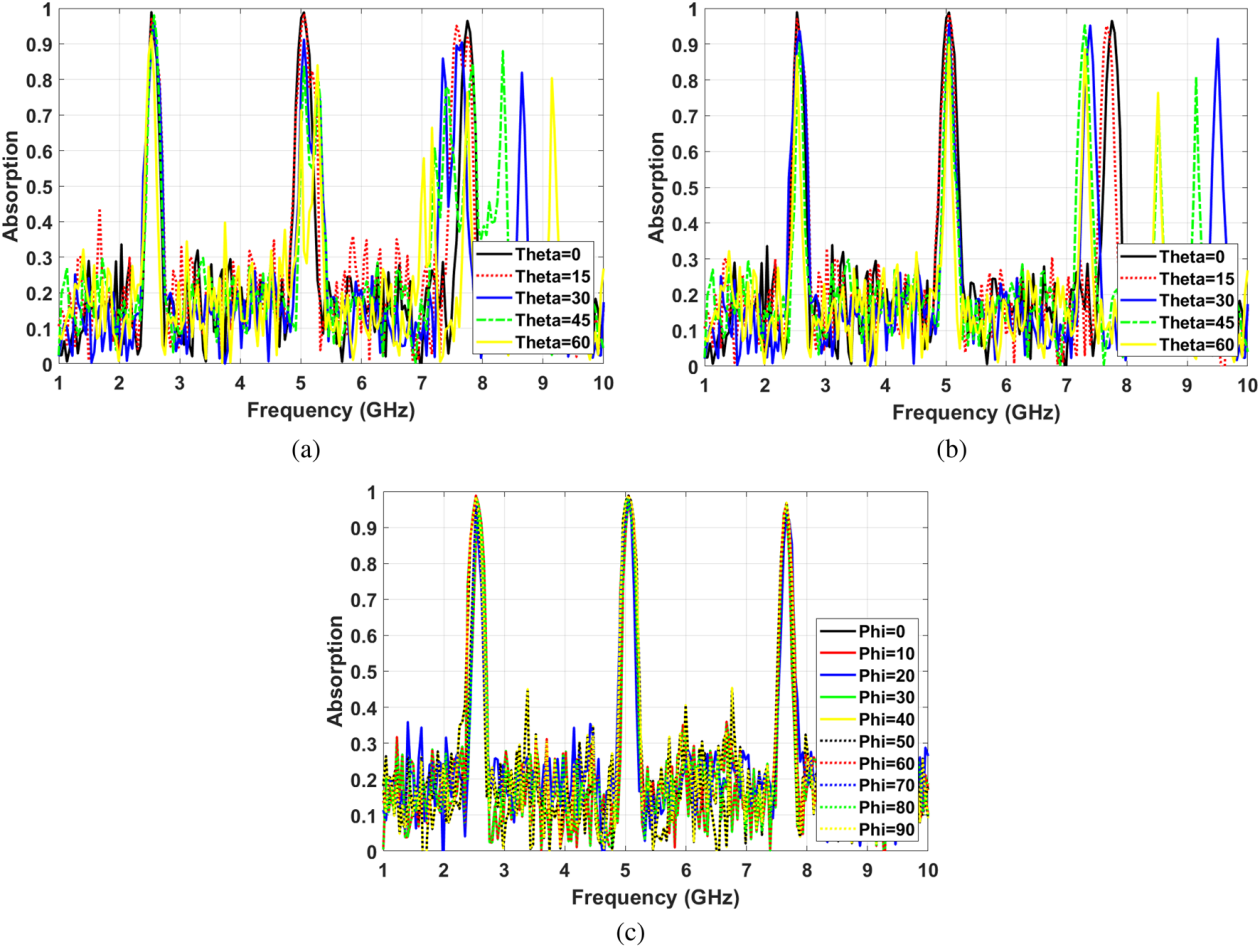
Angle insensitivity measurement results (**a**) Different incident angles for TE waves, (**b**) Different incident angles for TM waves, (**c**) Different polarization angles.

Figure 15a,b show the measured absorption at different incident angles from $\theta =0^{\circ }$ to $\theta =60^{\circ }$ under TE and TM polarized radiated electromagnetic waves, respectively. Moreover, Fig. 15c shows the absorption ratio’s measurement results at different polarization angles. The almost unchanged absorption ratio confirms the polarization angle insensitivity of the proposed MPA structure due to the symmetric resonator shape. Fig. 15 implies that measured absorption characteristics of the final structure have a significant correlation with simulation results.

**Figure 16:**
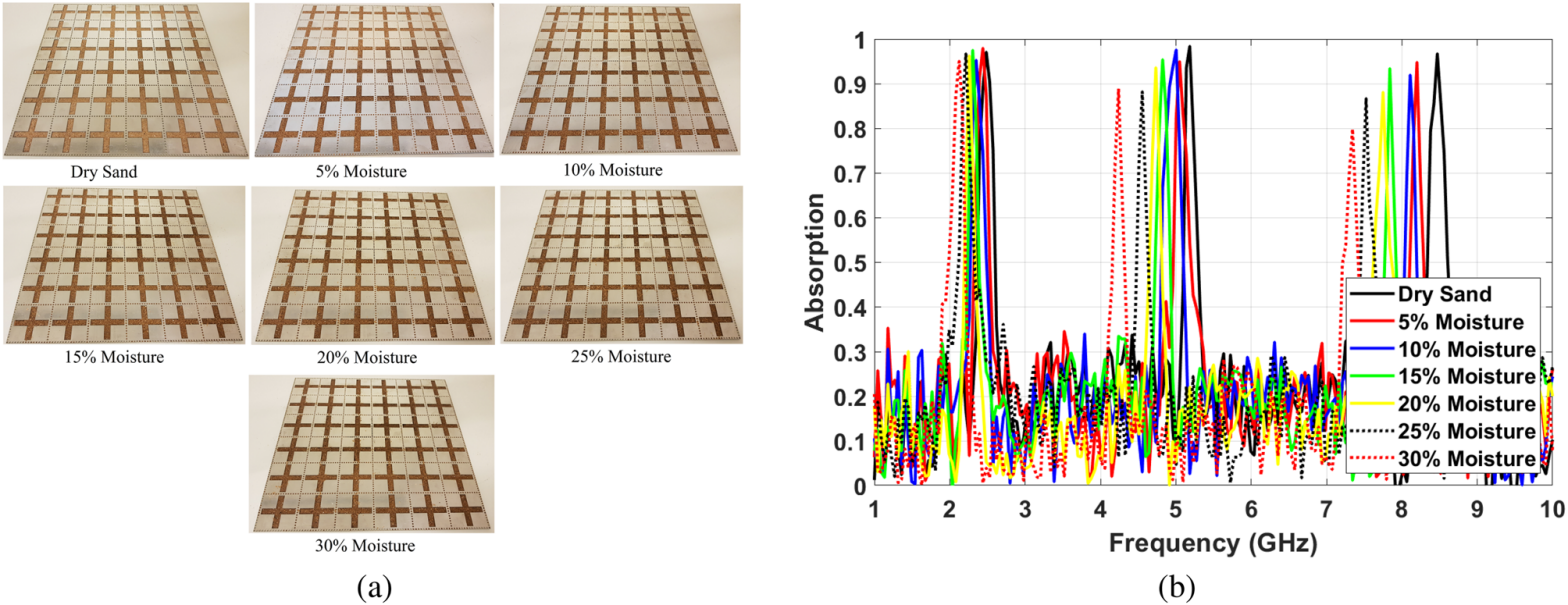
Measuring of sensing capability (**a**) Filling MPA channels by sand with different levels of moisture, (**b**) Changing absorption characteristics by varying the soil moisture.

After validating MPA’s performance with empty channels, sandy soil with different moisture content has been added to the structure. Different soil moisture levels have been achieved based on the “Soil survey standard test method soil moisture content”^59^ that has been released by the Department of Sustainable Natural Resources NSW Australia. The sand has been put in the oven initially to dry the sand. Then, the water has been added to provide sand with 5%, 10%, 15%, 20%, 25%, and 30% soil moisture levels. The soil moisture as a percentage of dry soil weight has been calculated according to the Eq. (16)^59^.

16 $$\begin{aligned} MC\%=\frac{W_2-W_3}{W_3-W_1} \times 100 \end{aligned}$$

where $W_1$, $W_2$, and $W_3$ are the weight of the tin container, weight of moist soil plus the tin container, and weight of dried soil plus tin container, respectively. Figure 16a shows the structure with filled channels by soil at different levels of moisture.

The changes in the absorption peak frequency are shown in Fig. 16b that strongly follow the simulation results. 360 MHz, 1 GHz, and 1.3 GHz downshift at first, second, and third absorption frequencies have been observed for changing moisture content in sand, respectively. Based on $S_{ave}$ equation, the sensitivities of fabricated structure are 60.79, 75.09, and 59.21 for changing the water content in the sand from 0 to 30%. Moreover, increasing the soil moisture level leads to the changing absorption ratio. As can be seen in Fig. 16b, the absorption ratio decreases from 98 to 91% at the first absorption frequency, from 98 to 85% at the second absorption frequency, and from 98 to 75% at the third absorption frequency when the sand moisture changes from 0 to 30%.

## Discussion

In this paper, a SIW cavity-based MPA has been introduced to implement a highly sensitive soil moisture sensor with an expected long operational lifetime. There is no need for an external power supply in the proposed sensor to sense soil moisture wirelessly. Furthermore, it can monitor the absorption characteristics from a distance by transmitting RF signals. Therefore, the proposed sensor is a promising candidate for remote sensing. Considering the effective medium theory and cavity principles, two perpendicular slots have been etched in the substrate to add the soil into the sensor structure. Furthermore, the SIW structure has been used to reduce the cost and complexity of the fabrication process. The sensing capability of the proposed sensor has been investigated for sand and loamy soil. The proposed sensor’s resolution is 10 MHz, 20 MHz, and 60 MHz per percent for increasing the sand moisture. For loamy, the resolution becomes 7.5 MHz, 22.5 MHz, and 52.5 MHz per percent at three resonance frequency, respectively. Besides the absorption frequency shift, the absorption ratio can be considered a sensing parameter due to the down-scaling by increasing the soil moisture level. Based on the simulation results, 17% and 52% drops have been observed for changing sand moisture from 0 to 30% at second and third resonance frequencies. On the other hand, 23% and 54% reductions have been shown for changing loamy soil moisture from 0 to 40%. Ultimately, an array of $6\times 6$ has been fabricated on the FR-4 substrate. Measurements follow the simulation result that confirms the correctness of the design procedure.

## Methods

**Figure 17:**
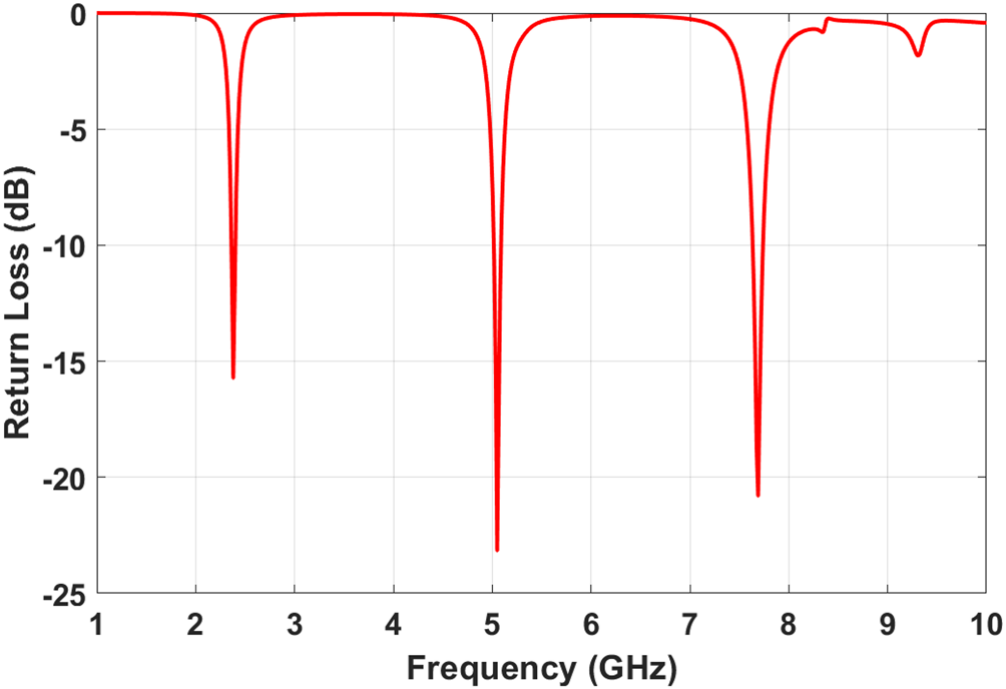
SIW-cavity MPA’s return loss before etching channels.

All simulations to design the proposed metamaterial absorber have been performed in commercial software CST STUDIO SUITE 2019 using a frequency-domain solver. The periodic boundary is applied for the unit cell to satisfy the metamaterial structure requirement. Parametric studies have been done under the normal incident angle to find optimum values for the proposed MPA parameters. Floqute port has been applied in simulation to facilitate changing incident and polarization angles to investigate structure sensitivity against different incident waves. The absorption characteristics have been extracted from Eq. (1) using return loss and transmission coefficient. Figure 17 shows the amount of reflected waves from MPA’s surface under normal incident waves before etching slots.

## Measurement

S-parameters have been used to calculate the absorption characteristics of the proposed MPA. These parameters were measured by implementing the described setup in Fig. 14b. A four ports ROHDE & SCHWARZ ZVA40 vector network analyzer (VNA) and two ROHDE & SCHWARZ HF907 dual-ridged horn reference antennas were used to perform measurements. One of the antennas has been connected to port one as a signal transmitter with a specified power of − 10 dBm. The other horn antenna performs to receive reflected signals from the MPA surface. Furthermore, the final setup was surrounded by wedge-trapped absorbing material to minimize the wave reflection in all directions. Before measuring S-parameter, the setup has been calibrated using a special calibration kit to neglect the effects of coaxial lines and connectors. The base level of transmission coefficient between antennas in the proposed setup must be specified by performing similar measurement scenarios using a metal plate with the same MPA size. These results were considered base level to compare with S-parameter and reduce the non-ideal testing setup’s noise. In order to measure absorptivity at different incident angles, two antennas were moved equally around a circle with a Radius of 1 m from the center of MPA. The $\theta$ is the angle between the perpendicular axis to the center of MPA and horn antenna in the azimuth plane. In the sequence of measuring absorptivity at different polarization angles, either horn antennas or MPA structure need to be rotated perpendicular around the perpendicular axis.
